# Hairy Cell Leukemia (HCL) Presenting with Joint Swelling: Case Report and Literature Review of a Rare Rheumatological Manifestation of a Hematological Disease

**DOI:** 10.7759/cureus.35912

**Published:** 2023-03-08

**Authors:** Vishwanath Anil, Venu Ganipisetti, Komal Harisinghani, Ashok Kumar Kanugula

**Affiliations:** 1 Internal Medicine, Wellstar Spalding Regional Hospital, Griffin, USA; 2 Internal Medicine, Presbyterian Hospital, Albuquerque, USA; 3 Internal Medicine, WellStar Health System-Splading Regional Hospital, Griffin, USA

**Keywords:** rheumatoid arthritis, braf v600e, disease mimics, felty’s syndrome, neutropenia, splenomegaly, arthritis, pancytopenia, joint swelling, hairy cell leukemia

## Abstract

Hairy cell leukemia (HCL) is a rare malignancy that primarily affects the bone marrow, peripheral blood, and spleen. The most common presenting features of HCL are splenomegaly or cytopenias causing fatigue, infections, or hemorrhagic manifestations. Symptoms involving the soft tissue or bone are rare. HCL can rarely present with immune-mediated polyarthritis. This presentation can be confused for other pathological entities, such as Felty’s syndrome, and can be differentiated from this with bone marrow biopsy. This case report looks into a rare presentation of HCL in which transient polyarthritis of the knees was the presenting symptom.

## Introduction

Hairy cell leukemia (HCL) is a rare mature lymphoid B-cell malignancy that affects the peripheral blood, spleen, and bone marrow [1]. With a median age of diagnosis of 55 and male predominance, it constitutes approximately 2% of all leukemias and less than 1% of all lymphoid neoplasms [2,3]. HCL classically presents with splenomegaly and pancytopenia [4]. Splenomegaly is a classic feature of HCL and a palpable spleen is often the sole finding on physical examination [5].

We present a case of HCL discovered after the patient presented with severe bilateral knee pain and swelling. Given the presence of polyarthritis, splenomegaly, and neutropenia, this presentation could be easily confused with Felty’s syndrome, a manifestation of rheumatoid arthritis (RA). Diagnosing HCL requires bone marrow biopsy and aspirate, along with immunophenotyping by flow cytometry [2,6]. Identification of “hairy” cells in the bone marrow smear and immunologic score based on the CD expression is required to conclude a diagnosis of HCL [1]. The HCL variant (HCL-V), a distinct pathology, is biologically different from HCL. This variant can be discerned by the difference in immunophenotyping and identifying the lack of BRAF mutation and monocytopenia [4]. HCL-V is associated with an infiltrative bone disease that can manifest as joint swelling; this phenomenon was ruled out in our case [7].

## Case presentation

A 55-year-old Caucasian male with a past medical history of uncontrolled hypertension and obesity presented to the emergency room (ER) with pain, redness, and swelling of bilateral knees, along with fatigue. His knee joint symptoms were insidious since onset, starting about three days before presentation and progressing rapidly. He first developed severe pain and swelling over his right knee, followed by similar symptoms in the left knee the next day. He described the severity of the knee pain as 10/10 and stated that he could not walk because of the pain, which prompted him to come into the ER. He also reported experiencing a subjective fever with chills about one day ago. He denied any recent weight loss or trauma.

Vital signs at presentation were a temperature of 97.8°F, blood pressure of 134/83 mmHg, a pulse of 101 beats/min, and a respiratory rate of 18 cycles/min. Physical examination at the time of admission was remarkable for bilateral knee swelling, erythema, and tenderness, and mild bilateral pedal edema.

His blood investigations revealed a white blood cell count of 1,860/µL (3,500-10,500/µL ) with an absolute neutrophil count of 130/µL (1,700-7,000/µL), hemoglobin of 8.6 g/dL (13.5-17.5 g/dL), hematocrit of 26.8% (39-50%), and platelet count of 43,00 /µL (150,000-450,000/ µL) (Table 1). We admitted the patient for further evaluation and management.

**Table 1:** Lab values during hospital course CBC: complete blood count; WBC: white blood cells; ANC: absolute neutrophil count

CBC parameters	Day 1	Day 2	Day 3	Day 4	Day 5	Day 6	Day 7	Reference range
WBC (/µL)	1,860	1,130	1,570	1,740	1,760	2,010	2,510	3,500-10,500
ANC (/µL)	130	110	70	90	80	90	100	1,700-7,000
Hemoglobin (g/dL)	8.5	6.8	7.8	7.9	8.3	8.6	9.5	13.5-17.5
Hematocrit (%)	26.8	21.2	23.8	24.3	25.2	26.1	29.5	39-50
Platelet count (× 1000 /µL)	43	39	36	39	35	37	39	150-450

**Figure 1:**
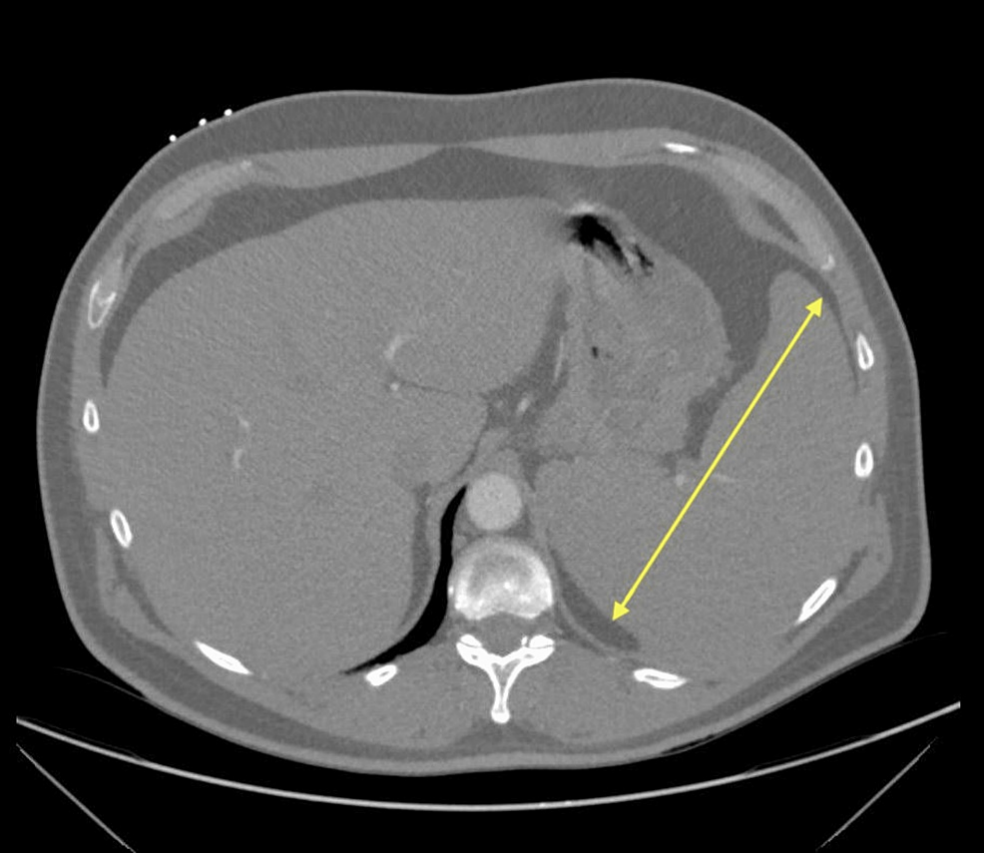
Initial CTA of chest showing splenomegaly, demonstrated with yellow line CTA: CT angiography

**Figure 2:**
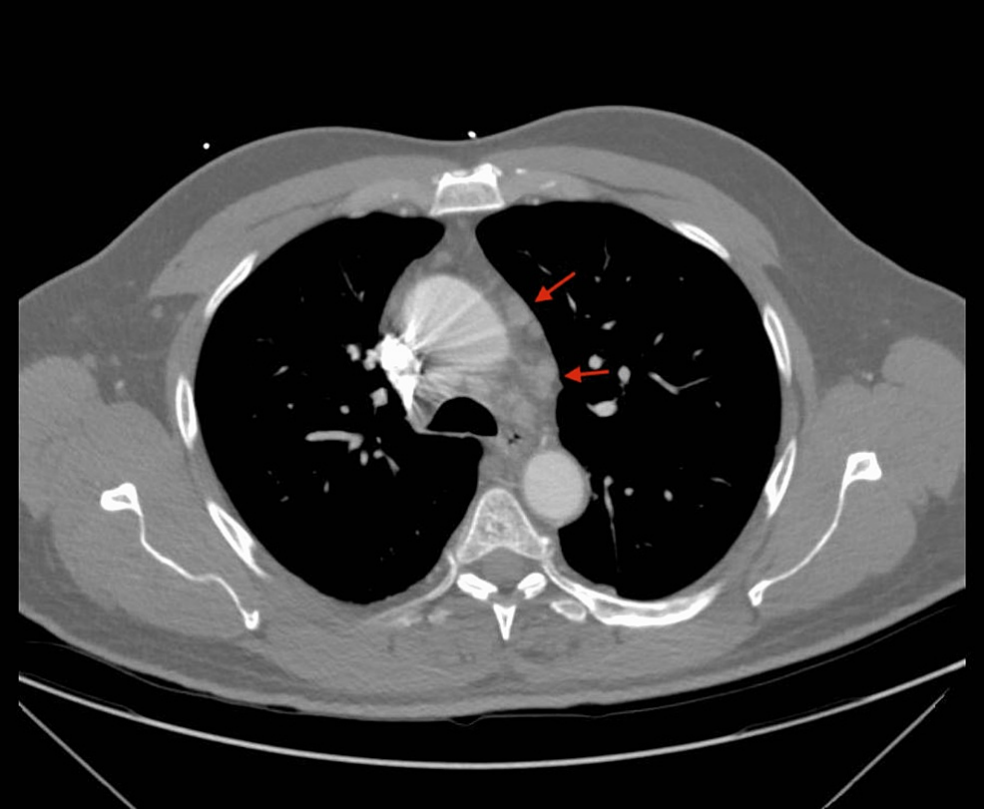
Initial CTA of chest showing mediastinal lymph nodes (red arrows) CTA: CT angiography

Upon admission, due to suspicion of sepsis in the setting of neutropenic fever, the patient was placed in isolation with neutropenic precautions and was started on empiric IV antibiotics (cefepime). Initial blood work also revealed a D-dimer of 3522 ng/mL, which prompted a CT angiogram (CTA) of the chest and venous doppler ultrasound of the legs. While the CTA helped rule out pulmonary embolism, it incidentally identified splenomegaly of 17.8 cm (Figure 1) and sub-centimeter mediastinal and hilar lymph nodes (Figure 2).

**Figure 3:**
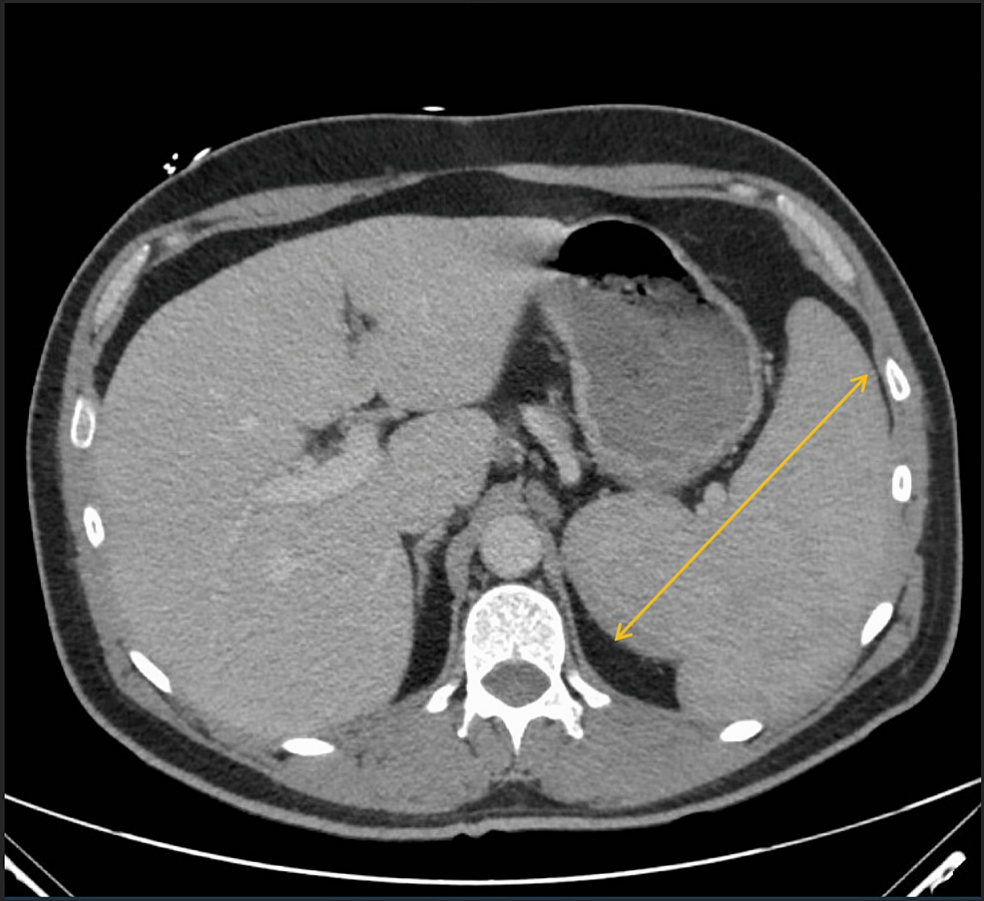
CT scan showing hepato-splenomegaly; yellow line demonstrates splenomegaly

CT of the abdomen and pelvis corroborated the splenomegaly (Figure 3) and revealed hepatomegaly and sub-centimeter left periaortic and superior mesenteric lymph nodes. A CT-guided bone marrow biopsy was done and the specimens obtained were sent for immunohistochemistry, flow cytometry, and cytogenetic study. The patient was noted to have a hemoglobin of 6.8 g/dL on the second day of admission and received a blood transfusion with one unit of packed red blood cells. Lactic acid, procalcitonin, and two blood culture sets were all negative. The patient’s knee pain and swelling improved significantly and were almost completely resolved spontaneously within the first three days of admission. Hence, additional rheumatological workup was not pursued. Apart from experiencing mild fatigue, the patient remained asymptomatic and afebrile throughout hospital admission.

**Figure 4:**
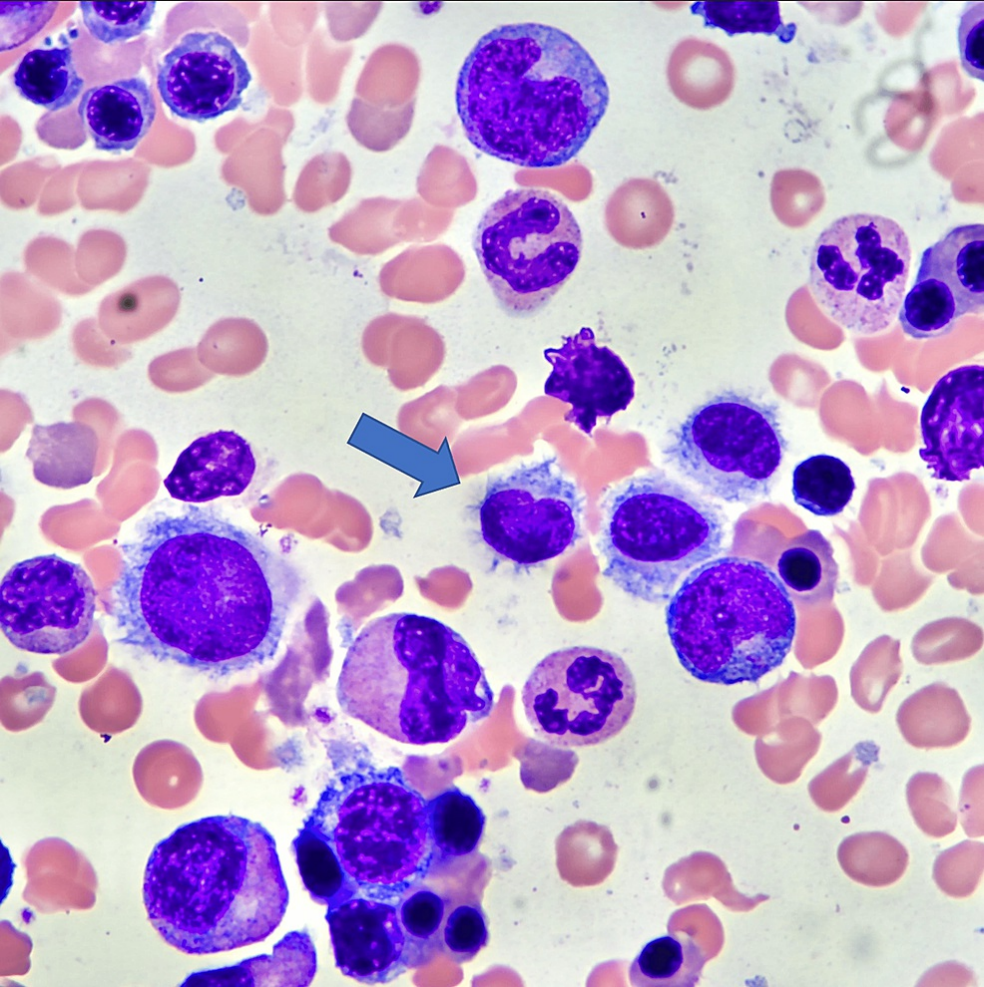
Bone marrow aspirate showing hairy cells (blue arrow)

The bone marrow smear (Figure 4) revealed some lymphoid cells that appeared to be HCL cells and, therefore, consistent with the diagnosis of HCL. CD 20 and BRAF V600E stains were positive. Flow cytometry study supported the impression of HCL with 29% lambda monotypic B cells positive for CD11c, CD19, CD20, CD25, and CD103. Eventually, we discharged the patient with an outpatient hematology-oncology follow-up. 

## Discussion

Classic HCL is a chronic lymphoproliferative disorder characterized by infiltrating malignant B cells with hair-like surface projections leading to progressive bone marrow failure [1]. HCL is known to be characterized by the presence of infections, splenomegaly, or cytopenia at the time of diagnosis. In 2011, with the use of whole-exome sequencing (WES), the BRAF V600E somatic mutation was identified in a patient with HCL. This mutation was subsequently identified in up to 70-100% of HCL cases [2]. The diagnosis is often confirmed with either tartrate-resistant acid phosphatase staining by cytochemistry or by a bone marrow biopsy revealing the classic hairy cells [5]. The flow cytometry markers that are positive in HCL include CD11c, CD25, CD103, and CD123, in addition to the typical B-cell markers, CD19, CD20, or CD22 [4]. The HCL-V accounts for about 10% of HCL cases [2]. HCL-V demonstrates no expression of CD25 and CD200, and the immunophenotype is that of a mature B-cell with positivity for CD103 and CD11c B-cell antigens [2,7]. When first described in 1958, HCL was associated with a dismal survival prognosis. By the 1990s, with the implementation of therapy with purine analogs such as cladribine, HCL had turned into one of the most successfully treated pathologies in cancer history [1].

Typical symptoms of HCL include abdominal discomfort or fullness (that results from massive splenomegaly), fatigue, weakness, infections, and bleeding manifestations [5]. The inability to obtain a bone marrow specimen with aspiration, i.e., a “dry tap”, is frequently reported with HCL [4]. A few patients may be asymptomatic at presentation [5]. A granulocyte count under 500/µL is associated with the development of life-threatening infection in about half the patients diagnosed with HCL [5].

Rarely has HCL also presented in the form of lymphomas in bones and soft tissues [8]. A retrospective review of 37 cases by Westbrook and Golde reported that six patients had joint symptoms [9]. Riambourg et al. reported that among the 27 cases of HCL, they managed, one presented with migrating arthralgia as the inaugural symptom [10]. This presentation has been attributed to either the hematological malignancy itself or an immune dysfunction [10]. This array of varying manifestations has often led to diagnostic uncertainty and potential underdiagnosis of this malignancy [8,10]. Furthermore, various case reports have described the presentation of RA with HCL. In the cases of immune-mediated inflammatory arthritis, the arthritic symptoms may occur before or after the onset of classic leukemic symptoms [11]. The case reported by L'Hirondel et al. describes short-lived polyarthritis symptoms resolved rapidly with alpha-2 interferon therapy [12]. Establishing a diagnosis is often complicated as HCL has also been observed to cause non-immune polyarthritis with synovial fluid identification of hairy cells as demonstrated by Zervas et al. [13]. HCL-V has been reported to develop painful joint disease due to leukemic infiltration causing bony expansion [7].

Felty’s syndrome, a manifestation of RA associated with neutropenia and splenomegaly, is usually caused by T-cell lymphoproliferative disorders [10]. This classic triad of symptoms makes it a primary differential diagnosis in our case. Interestingly, HCL is recognized as a rare predisposing factor for RA [10]. Zervas et al. describe a case of HCL later developing RA [13]. Taylor et al. report a case of seropositive inflammatory arthritis that developed in an HCL patient who received alpha interferon therapy [14]. There have been reports of Felty’s syndrome being complicated by the development of HCL [15]. Facchini et al. also reported that HCL can be a long-term sequela of RA [16]. 

Our patient presented with bilateral swelling and pain which elicited severe tenderness on physical exam. The symptom was severe enough to cause him to restrict ambulation. The patient’s BMI of 30.1 kg/m^2^ posed an additional challenge to palpate any splenomegaly on physical examination. Pancytopenia on blood work and the incidental finding of splenomegaly on the CTA raised suspicion of hematological malignancy and led to the subsequent diagnosis of HCL. Flow cytometry positive for CD25 plus the positive BRAF V600E stain result ruled out HCL-V. The spontaneous resolution of the swelling with conservative measures suggests that the symptom was probably a transient immune-mediated reaction rather than a direct manifestation of the malignancy such as leukemic infiltration.

## Conclusions

This case emphasizes the importance of recognizing the misleading rheumatologic manifestations of hematologic diseases, specifically highlighting the rare presentation of joint swelling as the initial symptom of HCL. The presentation of HCL with non-specific symptoms such as fatigue and joint swelling can lead to underdiagnosis if not thoroughly investigated. Therefore, clinicians should consider a broad differential diagnosis when evaluating patients with symptoms of arthritis in the setting of unexplained cytopenia.
